# Vertical Guidance Performance Analysis of the L1–L5 Dual-Frequency GPS/WAAS User Avionics Sensor

**DOI:** 10.3390/s100402609

**Published:** 2010-03-25

**Authors:** Shau-Shiun Jan

**Affiliations:** Institute of Civil Aviation, National Cheng Kung University, Tainan 70101, Taiwan; E-Mail: ssjan@mail.ncku.edu.tw; Tel: +886-6-275-7575 ext.63629; Fax: +886-6-238-9940

**Keywords:** aircraft navigation avionics sensor, GPS, GNSS Modernization, WAAS, MSAS, EGNOS, Galileo

## Abstract

This paper investigates the potential vertical guidance performance of global positioning system (GPS)/wide area augmentation system (WAAS) user avionics sensor when the modernized GPS and Galileo are available. This paper will first investigate the airborne receiver code noise and multipath (CNMP) confidence (*σ_air_*). The *σ_air_* will be the dominant factor in the availability analysis of an L1–L5 dual-frequency GPS/WAAS user avionics sensor. This paper uses the MATLAB Algorithm Availability Simulation Tool (MAAST) to determine the required values for the *σ_air_*, so that an L1–L5 dual-frequency GPS/WAAS user avionics sensor can meet the vertical guidance requirements of APproach with Vertical guidance (APV) II and CATegory (CAT) I over conterminous United States (CONUS). A modified MAAST that includes the Galileo satellite constellation is used to determine under what user configurations WAAS could be an APV II system or a CAT I system over CONUS. Furthermore, this paper examines the combinations of possible improvements in signal models and the addition of Galileo to determine if GPS/WAAS user avionics sensor could achieve 10 m Vertical Alert Limit (VAL) within the service volume. Finally, this paper presents the future vertical guidance performance of GPS user avionics sensor for the United States’ WAAS, Japanese MTSAT-based satellite augmentation system (MSAS) and European geostationary navigation overlay service (EGNOS).

## 1. Introduction

Presently, the only fully operational Global Navigation Satellite System (GNSS) is the Global Positioning System (GPS), which was developed, implemented, and is operated by the US Department of Defense (DoD) to provide position, velocity, and time information to users worldwide [1,2]. GPS currently has two operational signals, L1 at a center frequency of 1575.42 MHz and L2 at a center frequency of 1227.6 MHz. The L1 signal is modulated by both a 10.23 MHz clock rate precision P(Y) code and by a 1.023 MHz clock rate C/A code. On most GPS satellites, the L2 signal is modulated by only the P(Y) code. The P(Y) code is for authorized users and the C/A code is for civil users [2], therefore, the current civil users can only access the L1 C/A. A stand-alone GPS user can typically estimate location with an accuracy of 10 meters [1].

The GNSS is undergoing substantive changes that will enhance its capabilities in all applications. This future GNSS includes three key elements:

- *GNSS Augmentation Systems,* which monitor the GNSS satellites and provides error bounds to safety-critical users in real time. The first of these GNSS Augmentation Systems is the Wide Area Augmentation System (WAAS) [3] in the United States, which achieved Initial Operational Capability (IOC) on July 10, 2003. WAAS currently monitors the GPS constellation to provide differential corrections and a certified level of integrity. The corrections will improve the accuracy of the system, and more importantly, the integrity will open the door for widespread aviation use. There are many countries working on similar systems. The European Space Agency (ESA) is working on the European Geostationary Navigation Overlay Service (EGNOS) which became operational on October 01, 2009, and Japan deploys the MTSAT-based Satellite Augmentation System (MSAS), which was commissioned on September 27, 2007. Taiwan Civil Aeronautics Administration (CAA) plans to replace the current ground based assisting navigation equipment with the augmented GNSS within the next few years.
- *GPS modernization* will add two new civil signals to the GPS positioning and timing services. A second civil signal (L2C) will be added at the second GPS frequency, L2, at 1227.6 MHz. A third civil signal, L5, will be added at a lower frequency 1176.45 MHz [4]. Both L1 and L5 are in Aeronautical Radio Navigation Services (ARNS) bands, and are for safety-of-life services. On the other hand, L2 is not in an ARNS band and is for non-safety critical applications. The use of these additional frequencies is expected to enhance the performance of GPS. These three civil GPS signals will improve the performance of the GPS receiver, and will enable a new algorithm to estimate and mitigate the ionospheric delay, which currently is the largest obstacle for the GPS to become the primary navigation aid in civil aviation.
- *New GNSS Constellations* are under development by many countries worldwide, for instance, Galileo is the Europe’s contribution to a global navigation satellite infrastructure [5]. Using both infrastructures (GPS and Galileo) in a coordinated fashion offers real advantages in terms of availability and in terms of security, should one of the two systems becomes unavailable. Similarly, China is working on the Compass system and Russia is updating the GLONASS. This paper will use Galileo as an example to evaluate the performance improvement from the additional GNSS constellation.

While the Federal Aviation Administration (FAA) WAAS works well under nominal conditions, it is susceptible to the ionospheric disturbance and to the satellite outages. Because of the addition of the new frequencies (L2 and L5) and the new constellation (Galileo), WAAS should be modernized to take advantage of the modernized GPS and Galileo. Therefore, the objectives of this paper are to assess the following two questions. First, can WAAS achieve 20 m Vertical Alert Limit (VAL) (*i.e.*, Required Navigation Performance (RNP) of APV II) [6] over conterminous United States (CONUS) with improved modeling and Final Operational Capability (FOC) infrastructure? Second, can WAAS be a CAT I (12 m VAL, RNP of CAT I) [6] system or possibly achieve 10 m VAL over CONUS with the additional new civil frequencies and satellites from Galileo?

Accordingly, this paper is organized as follows: Section 2 discusses the system analysis assumptions. The performance of the upgraded WAAS is discussed in Section 3. Section 4 discusses the performance of the L1–L5 dual-frequency WAAS. The possible benefit from the improved signal model is discussed in Section 5. In Section 6, the dual-mode WAAS (GPS and Galileo) is investigated. Section 7 presents a summary and some concluding remarks.

## 2. Performance Analysis of WAAS

The availability of WAAS is determined by the confidence bounds on position errors and the satellite geometry. The computation of confidence estimates for the corrections to various error sources are defined in the WAAS Minimum Operational Performance Standard (MOPS) [7]. The error due to ionospheric delay and satellite errors will be corrected according to the WAAS MOPS as well, and then the local errors such as error due to tropospheric delay and user receiver noise and multipath errors will be removed by a standard model [7]. The corrected range measurements are used to compute GPS position and receiver clock errors using weighted least squares as follows,

(1) $$\hat{x}={\left(G^{T}WG\right)}^{-1}G^{T}Wy$$

where

*x̂* is the position and clock errors,
*G* is the observation matrix,
*W* is the weighting matrix for the measurement, and
*y* is the corrected range residual vector.

The weighting matrix, *W*, is a diagonal matrix and the inverse of the *i^th^* diagonal element is given by the variance for the corresponding satellite, $\sigma_{i}^{2}$ which is calculated in Equation (2).

(2) $$\sigma_{i}^{2}=\sigma_{i,\mathrm{flt}}^{2}+\sigma_{i,\mathrm{UIRE}}^{2}+\sigma_{i,\mathrm{air}}^{2}+\sigma_{i,\mathrm{tropo}}^{2}$$

where

$\sigma_{i,\mathrm{flt}}^{2}$ is the fast and long-term degradation confidence, which is the confidence bound on satellite clock and ephemeris corrections [7],
$\sigma_{i,\mathrm{UIRE}}^{2}$ is the user ionospheric range error confidence, which is the confidence bound on ionospheric delay corrections [7],
$\sigma_{i,\mathrm{air}}^{2}$ is the airborne receiver error confidence, which is the confidence bound on aircraft user receiver error [7], and
$\sigma_{i,\mathrm{tropo}}^{2}$ is the tropospheric error confidence, which is the confidence bound on residual tropospheric error [7].

As a result, the inverse of *W* can be written in Equation (3)

(3) $$W^{-1}=\left[\begin{array}{cccc} \sigma_{1}^{2} & 0 & \cdots & 0\\ 0 & \sigma_{2}^{2} & \cdots & 0\\ \vdots & \vdots & \ddots & \vdots \\ 0 & 0 & & \sigma_{N}^{2} \end{array}\right]$$

The variance of the vertical position estimate is the third diagonal element of the position estimate covariance matrix,

(4) $$d_{3,3}={\left[{\left(G^{T}WG\right)}^{-1}\right]}_{3,3}$$

where

*d*_3,3_ is the variance of the vertical position estimate.

The VPL (Vertical Protection Level) is

(5) $${VPL}_{WAAS}=K_{V,PA}d_{3,3}$$

where, *K_V,PA_* equals 5.33. This is a multiplier on the standard deviation of the vertical error such that the VPL is only exceeded at most one time in ten million (10^−7^), the tolerable probability of HMI (Hazardously Misleading Information), provided that the error distribution is a zero mean Gaussian [8]. The protection level calculation is specified in the WAAS MOPS Appendices A and J [7]. The VPL is very important and will be used to determine the availability of WAAS.

**Figure 1.**
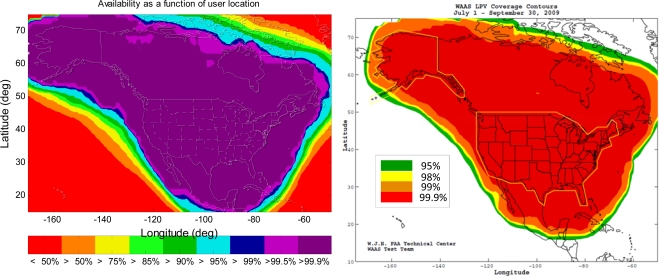
The 99.9% LPV availability contours of the current WAAS. The left plot is the MAAST simulation result (a 24 hour average of LPV availability using the actual almanac data of August 15th, 2009), and the right plot is the actual performance of the WAAS (a three month average of LPV availability, July 1st to September 30th, 2009).

The simulation tool in this paper is the MATLAB^®^ Algorithm Availability Simulation Tool (MAAST) [9], a publicly available software tool co-developed by the author, which can be customized to simulate the WAAS confidence estimation algorithms and evaluate the effect of service availability with algorithm changes. Note that MAAST is available in [10]. MAAST assumes 100% asset reliability (*i.e.*, no satellite, reference station, or communication failures), and is therefore somewhat optimistic, and one would expect actual performance to be slightly worse. As indicated in [11] and Figure 1, the simulation result of MAAST (the left plot of Figure 1) is very similar to the actual performance of the WAAS (right plot of Figure 1). When comparing the 99.9% Localizer Performance with Vertical guidance (LPV) [6] availability contours in Figure 1 (*i.e.*, the dark purple region in the left plot and the red region in the right plot), the MAAST simulation result is more optimistic and less smooth than the actual long-term average WAAS result (July 1st to September 30th, 2009), but it could determine the limits of availability to within a few degrees of the correct location [11]. MAAST is intended for use as a fast, accurate, and highly customizable experimental test bed for WAAS algorithm development. The remainder of this paper shows only the VPL simulation results, because in general GPS has more difficulties meeting the vertical guidance requirement.

## 3. WAAS System Upgrade

WAAS contains three segments: control segment, space segment, and user segment [3]. The WAAS control segment includes a geographically distributed set of GPS L1 (1575.42 MHz) and L2 (1227.6 MHz) dual-frequency receivers at precisely known reference locations. These receivers continuously monitor all of the GPS satellites, and are called wide area reference stations (WRSs). These WRSs send raw GPS measurements back to the wide area master stations (WMSs) where vector corrections are generated. These vector corrections consist of the satellite ephemeris and clock errors, and a grid of ionospheric delays. The data stream also includes confidence bounds for the corrections and “Use/Do-Not-Use” messages to provide integrity. These messages are then passed to the WAAS space segment through a Ground Uplink System (GUS). The current WAAS space segment contains two geostationary satellites (GEOs). These are the PRN-135 (Intelsat, 133° west) and PRN-138 (Telesat, 107.3° west). The GEOs broadcast the integrity messages and vector corrections on the same frequency as GPS L1 to user equipment (*i.e.*, WAAS avionics sensor). These GEOs also act as additional ranging sources to enhance service availability.

**Figure 2.**
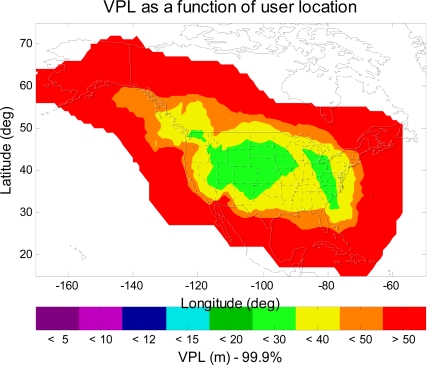
The 99.9% VPL contour of IOC WAAS. The VPL values in CONUS are greater than 20 m, and some places are higher than 50 m (LPV VAL).

Figure 2 is the MAAST simulation result for the VPL contour of IOC (Initial Operational Capability) WAAS. The important parameters used in the simulation are: the twenty four standard GPS satellites constellation defined in the WAAS MOPS, one-degree user grid within the service volume, and thirty-second time steps over a twenty four hour simulation period. To be consistent and for ease of comparison in the results, these simulation parameters of MAAST in the remainder of this paper will remain the same. Figure 2 indicates that a user avionics sensor at each specific location had a VPL equal to or below the value indicated by the color bar 99.9% of the time. In this paper, we are interested in the following aviation navigation services: LPV (VAL (Vertical Alert Limit) = 50 m), APV II (VAL = 20 m), and CAT I (VAL = 12 m) [6]. As shown in Figure 2, most of CONUS has LPV service of very high availability (equal to 99.9% or greater). However, there are some regions do not meet the LPV requirement (VPL > 50 m (LPV VAL)).

WAAS has been upgraded to better meet the needs of civil aviation users. Since IOC, 13 new reference stations have been added to the WAAS network, with five of them added in Mexico, four in Canada, and another four added in Alaska. These new reference stations expand the WAAS service coverage. By adding new reference stations to Mexico and Canada, WAAS becomes an international system.

The operating principle of ionospheric correction of the WAAS is to employ a set of reference stations to monitor the GPS signals so as to come up with corrections. Similar to the Nyquist sampling theorem, a key to the success of the approach is that the reference stations and ionospheric grid points (IGPs) must be dense enough to account for the variation of the ionosphere. Thus, the estimation of the ionospheric delay would be benefited from more reference stations. In addition, the IGP mask limits the WAAS precision approach and landing service region, because WAAS users must obtain the real-time ionospheric corrections in order to perform the vertical guidance [7]. The IGP mask around Alaska and Canada will be expanded to gain more samples of ionosphere observation. The WAAS ionosphere working group is working on the expansion of the IGP mask, and an example of the expanded IGP mask will be used in this paper for the WAAS service volume analysis.

**Figure 3.**
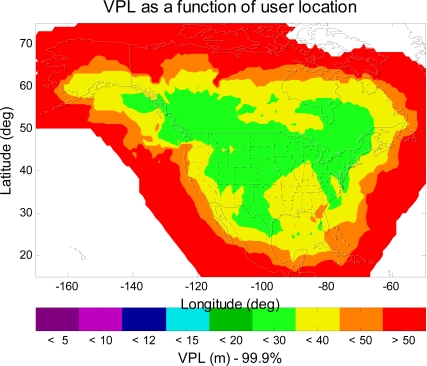
The 99.9% VPL contour of the upgraded WAAS. The VPL values in all of CONUS and in most of Alaska are less than 50 m (LPV VAL).

**Figure 4.**
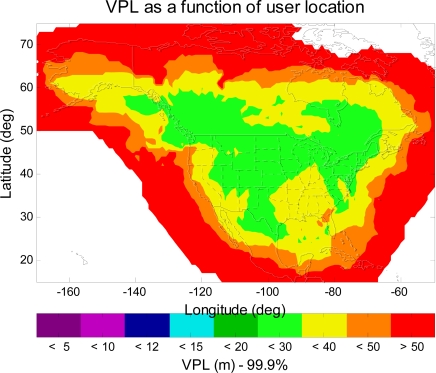
The 99.9% VPL contour of the upgraded WAAS with more IGP in Alaska. In comparison with Figure 3, the LPV service coverage in Alaska is improved.

Figure 3 shows the VPL contour of the upgraded WAAS with 38 reference stations, two GEOs and an IGP mask with expansion around Alaska and Canada. As can be seen in Figure 3, users in all of CONUS, in most of Alaska and Canada, will have LPV service with 99.9% or greater availability (VPL < 50 m (LPV VAL)). Figure 4 shows another VPL contour of the same upgraded WAAS, but a different IGP mask is used in this simulation. This IGP mask has more IGP in the northeast region of Canada and in the northwest region of Alaska than the expanded IGP mask used in Figure 3. As can be seen in Figure 4, the 99.9% LPV service coverage is further extended to the northwest region of Alaska. However, the LPV service coverage is not significantly improved over that of Figure 3. That is because we do not increase the number of WAAS reference station in the same region. In other words, there are not enough ionosphere observations to support the additional IGP. Therefore, one could expect more improvement in the LPV service coverage in the northeast region of Canada and in the northwest region of Alaska provided that there are new additional reference stations. Figure 4 however shows that we could gain some improvement in the LPV service coverage by modifying the Grid Ionospheric Vertical Error (GIVE) algorithms [7] with more IGP.

**Figure 5.**
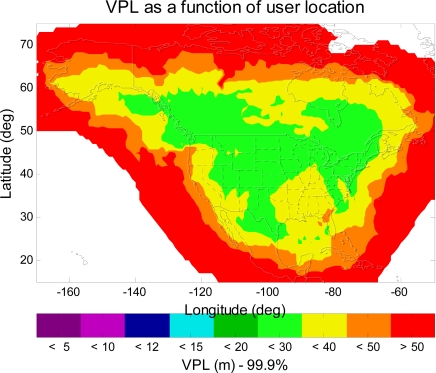
The 99.9% VPL contour of the upgraded WAAS with extended IGP and three GEOs. In comparison with Figure 4, the LPV service coverage in the north region of Canada is slightly improved.

The FAA also plans to add a new geostationary satellite (PRN-133) in 2010, which will be at 98°W. The new additional GEO is to ensure that all users in WAAS service volume will have at least two GEOs in view, and the new additional GEO could improve the satellite geometry for better positioning and continuity. Figure 5 shows another VPL contour of the same upgraded WAAS, but three GEOs are used in this simulation. As can be seen in Figure 5, the 99.9% LPV service coverage is further extended to the north region of Canada. The additional GEO improves the geometry for the position estimation.

## 4. L1–L5 Dual-Frequency WAAS

As described in the first section, GPS will add a new civil frequency, L5 at 1176.45 MHz, in the ARNS band. This new civil GPS signal combined with current L1 will improve the performance for GPS users by enabling them to estimate and mitigate the ionosphere delay. Recall that ionospheric delay currently is the largest obstacle for the GPS to become the primary navigation aid in civil aviation.

An L1–L5 dual-frequency GPS user avionics sensor can estimate the ionospheric delay directly (*i.e.*, no ionosphere correction needed from WAAS) and then subtract this estimation from the pseudorange measurements. This direct use of the L1–L5 dual-frequency will be more accurate and offer higher availability [12–14]. WAAS should be modernized to take advantage of these new and stable signals. The major changes will be in the WAAS ionosphere model (algorithm). The detailed changes of the L1–L5 dual-frequency WAAS algorithms are specified in [13]. For an L1–L5 dual-frequency GPS/WAAS user avionics sensor, the weighting matrix, *W*, is a diagonal matrix and the inverse of the *i^th^* diagonal element is given by the variance for the corresponding satellite, $\sigma_{i,\text{dual}}^{2}$ as calculated in Equation (6) [13]:

(6) $$\sigma_{i,\text{dual}}^{2}=\sigma_{i,\mathrm{flt}}^{2}+\sigma_{i,\mathrm{air},L1-L5}^{2}+\sigma_{i,\mathrm{tropo}}^{2}$$

where

- $\sigma_{i,\mathrm{flt}}^{2}$ and $\sigma_{i,\mathrm{tropo}}^{2}$ are defined in the same manner as in Equation (2), and
- $\sigma_{i,\mathrm{air},L1-L5}^{2}$ is the L1–L5 dual-frequency airborne receiver error confidence, which is the confidence bound on ionospheric-free receiver measurements for an L1–L5 dual-frequency GPS/WAAS user avionics sensor and is derived in [13]. Note: Because the calculation of $\sigma_{i,\mathrm{air},L1-L5}^{2}$ already considers both the L1–L5 dual-frequency user ionosphere range error confidence and the airborne multipath and noise error confidence, there is no need for additional terms accounting for these errors in Equation (6) [13].

**Figure 6.**
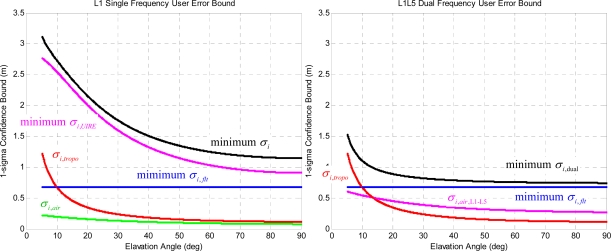
The minimum user error components as a function of satellite elevation angle. The left figure is the minimum L1 single frequency GPS/WAAS user error components, and the right figure is the minimum L1–L5 dual-frequency GPS/WAAS user error components.

Figure 6 shows the comparison of the error components between the L1 single frequency GPS/WAAS user (*i.e.*, Equation (2)) and the L1–L5 dual-frequency GPS/WAAS user (*i.e.*, Equation (6)). The specific numbers used in the calculations are based on the nominal observations of WAAS. For the L1 single frequency GPS/WAAS user avionics sensor, the minimum *σ*_*i*,*flt*_ term is based on the minimum User Differential Range Error (UDRE) [7] of 2.25 m [11], the minimum *σ*_*i*,*UIRE*_ term is based on the minimum GIVE value of 3 m [7], the *σ*_*i*,*air*_ term uses the Airborne Accuracy Description (AAD-B) model defined in [7], and the calculation of *σ*_*i*,*tropo*_ term is defined in [7]. For the L1–L5 dual-frequency GPS/WAAS user avionics sensor, the *σ*_*i*,*flt*_ and *σ*_*i*,*tropo*_ terms are identical to those of the L1 single frequency GPS/WAAS user, and the *σ*_*i*,*air*,L1–L5_ term is defined in [13] which is larger than *σ*_*i*,*air*_ but is significantly smaller than *σ*_*i*,*UIRE*_. As indicated in Figure 6, the *σ*_*i*,*flt*_ term is the dominant error component for the L1–L5 dual-frequency GPS/WAAS user avionics sensor.

**Figure 7.**
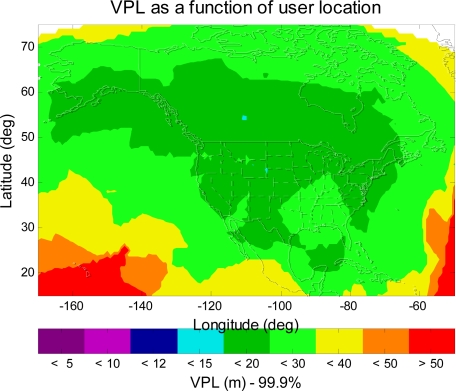
The 99.9% VPL contour of the L1–L5 dual-frequency GPS/WAAS user avionics sensor. The VPL values in most of CONUS, Alaska, and Canada are less than 20 m (APV II VAL).

Figure 7 shows the VPL contour of the L1–L5 dual-frequency GPS/WAAS user. As shown in Figure 7, the VPL values are less than 20 m (APV II VAL) in most of CONUS, Alaska, and Canada. This is a significant improvement from Figure 3, 4 or 5 (single frequency WAAS), however, the VPL contour shown in Figure 7 still falls short of meeting the CAT I requirement (CAT I VAL = 12 m). In the next section, this paper will seek additional improvement that could be possible made in the dual-frequency WAAS user avionics sensor algorithm, and examine if the dual-frequency WAAS could meet CAT I requirement (VAL = 12 m).

## 5. Possible Signal Model Improvement

**Figure 8.**
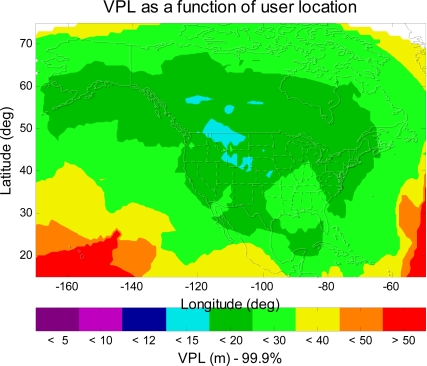
The 99.9% VPL contour of the dual-frequency GPS/WAAS user with an improved signal model. The VPL values could not meet the CAT I requirement.

The new civil frequency at L5 will have more signal power than the current civil signal at L1 [1]. The higher civil signal power will enable users to acquire GPS satellites earlier for smoothing before using them for position estimation. Thus, the floor of the residual user receiver noise and multipath error ( $\sigma_{i,\mathrm{air},L1-L5}^{2}$ in Equation (6)) would be lower than the current model [15]. An upper bound of the benefit is found by setting the residual user receiver noise and multipath error to be *zero*. The MAAST simulation result is shown in Figure 8. As can be seen in Figure 8, however, the VPL values still does not meet the CAT I requirement (CAT I VAL = 12 m), despite improvement of some region to VPL < 15 m.

For an L1–L5 dual-frequency WAAS user avionics sensor, the dominant term in the confidence calculation of Equation (6) is the $\sigma_{i,\mathrm{flt}}^{2}$, which is the confidence bound on satellite clock and ephemeris corrections. However, any reduction in the confidence calculation of $\sigma_{i,\mathrm{flt}}^{2}$ will require a substantial change in the WAAS UDRE (User Differential Range Error) algorithms [7].

## 6. Dual-Mode WAAS (GPS + GALILEO)

Galileo is the Europe’s contribution to a global navigation satellite infrastructure. Galileo is expected to reach the Full Operational Capability (FOC) in 2013. In June 2004, the European Union and the United States signed an agreement to envisage the compatibility and interoperability of GPS and Galileo. In other words, one will be able to calculate a position with the same receiver from any of the satellites in both systems. It will make the user more robust to the loss of the satellites.

**Figure 9.**
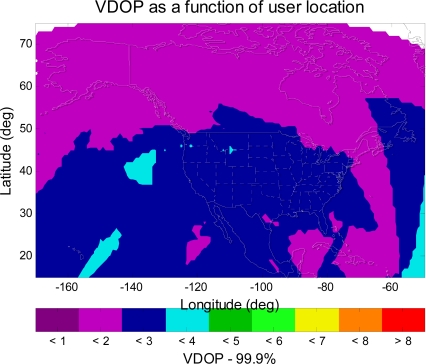
The 99.9% VDOP contour for WAAS with GPS alone. The VDOP values are from 1–4 in CONUS, and the VDOP values are from 1–2 in Alaska.

**Figure 10.**
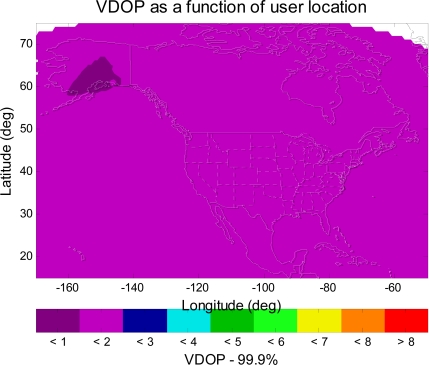
The 99.9% VDOP contour for WAAS with GPS and Galileo. The VDOP values are from 1–2 in all of CONUS, and in all of Alaska.

WAAS should also take advantage of the new satellite constellation. Therefore, WAAS should provide corrections to Galileo as well as GPS. In the service volume analysis, this paper treats Galileo satellites the same as GPS satellites but in different orbits. With a combined GPS and Galileo constellation, the MAAST simulation shows that the number of satellites in view over a twenty four hour period is more than twenty for each simulation time step (five minutes). This is about twice the number with GPS only. One could expect significant improvement in the geometry for the position estimation. Figure 9 and Figure 10 show the VDOP (Vertical Dilution of Precision) [1,2] for WAAS with GPS alone and for WAAS with GPS and Galileo, respectively. In comparison with Figure 9, Figure 10 shows a significant improvement in VDOP. As can be seen in Equation (4), generally good geometries (small VDOP) as well as good confidence bounds (small *σ_i_*) are required to obtain high availability [1].

**Figure 11.**
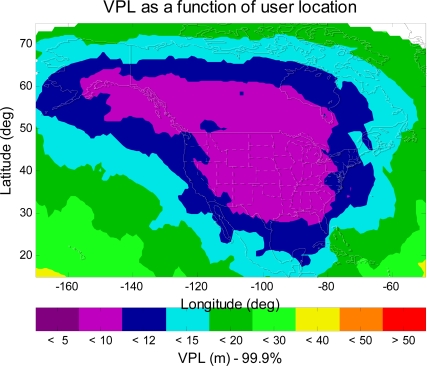
The 99.9% VPL contour of the dual-mode (GPS + Galileo) and L1–L5 dual-frequency WAAS user avionics sensor. The VPL values in most of CONUS, Alaska, and Canada are less than 12 m (CAT I VAL). Importantly, The VPL values are less than 10 m in most of CONUS and Canada.

Figure 11 shows the VPL contour for the dual-mode (GPS + Galileo) and dual-frequency (L1 + L5 and/or E1 + E5) WAAS user avionics sensor. As shown in Figure 11, the VPL values are less than 12 m (CAT I VAL) in most of CONUS, Alaska, and Canada. Furthermore, as can be seen in Figure 11, the VPL values are less than 10 m in most of CONUS, in most of Canada, and in some of Alaska. In other words, it is possible for WAAS to achieve 10 m VAL (a more stringent landing requirement) with new additional civil signals and additional satellites from Galileo. The VPL is a very important criterion to evaluate the performance of WAAS, and many resources have been focused on reducing this term. The goal is not only to meet the current LPV requirement but also be capable to meet the more stringent required navigation performance (RNP), such as LPV 200 (VAL = 35 m) and CAT I (VAL = 12 to 10 m) requirements. For instance, the FAA’s performance goals of WAAS are first to provide full LPV service by September 2008, then provide LPV 200 service in 2009–2013, and finally provide dual-frequency WAAS service (possible Category I) in 2014–2028. This paper discusses the modernized aviation L1–L5 dual-frequency GNSS/WAAS user, thus, it is necessary to explore all possible techniques to gain better VPL performance (*i.e.*, reduction in VPL). As a result, it is important to find the future architectures which allow aviation users to enhance the performance of a GPS/WAAS approach and landing system.

**Figure 12.**
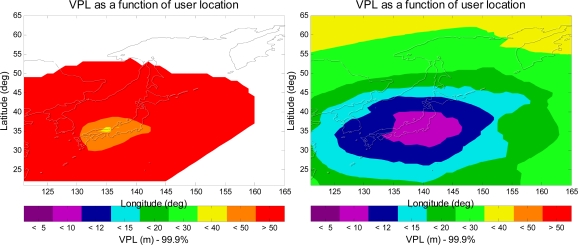
The 99.9% VPL contours of the MSAS user avionics sensor. The left figure is for the current MSAS user avionics sensor, and the right figure is for an L1–L5 dual-frequency MSAS avionics sensor with GPS and Galileo.

**Figure 13.**
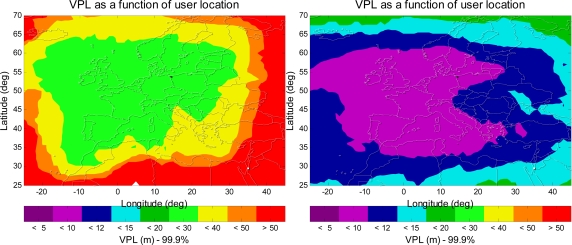
The 99.9% VPL contours of the EGNOS user avionics sensor. The left figure is for the current EGNOS user avionics sensor, and the right figure is for an L1–L5 dual-frequency EGNOS avionics sensor with GPS and Galileo.

This paper extends the same analysis to investigate the aviation navigation performance of Japanese MSAS and European’s EGNOS. Figure 12 presents the VPL contours for Japanese MSAS. The left figure shows the VPL contour for the current MSAS user avionics sensor, and the right figure shows the VPL contour for an L1–L5 dual-frequency MSAS avionics sensor with GPS and Galileo. As shown in the figure, the aviation navigation performance of the MSAS avionics sensor is significantly improved by the use of L1–L5 dual-frequency and Galileo. Figure 13 shows the VPL contours for ESA EGNOS, and the aviation navigation performance of the EGNOS avionics sensor is also greatly improved by the use of L1–L5 dual-frequency and Galileo. As a result, it is a significant improvement for MSAS and EGNOS with the new additional civil signals and additional satellites from Galileo.

## 7. Conclusions

This paper investigated the vertical guidance performances of different phases of GPS/WAAS user avionics sensor for the next 15–20 years. First, this paper showed the performance of the IOC WAAS user avionics sensor. This paper then showed that upgraded WAAS could provide LPV (50 m VAL) service to the GPS/WAAS user avionics sensors in all of CONUS and in most of Alaska with 38 reference station, three GEOs, and an expanded IGP mask. Second, with the second civil signal (L5) WAAS could provide APV II (20 m VAL) service to the GPS/WAAS user avionics sensors in most of CONUS, Alaska, and Canada. Because the ionosphere is currently the largest error source on GPS, the second civil frequency provided a significant improvement on the GPS/WAAS user avionics sensor performance. An L1–L5 dual-frequency user avionics sensor could estimate the ionospheric delay directly and then subtract this estimation from the pseudorange observations. This direct use of the dual-frequency signals will be more accurate and offer higher availability. Because the new civil signals will have stronger power than current signal, this paper therefore lowered the floor of the residual user receiver noise and multipath error to evaluate if the GPS/WAAS user avionics sensor could meet 12 m VAL (CAT I). Unfortunately the L1–L5 dual-frequency GPS/WAAS user avionics sensor with the enhanced signal model could not meet the CAT I requirement (VPL > 12 m VAL).

This paper then analyzed the VDOP improvement from the new satellite constellation composed of the combination of GPS and Galileo. It is shown that the dual-mode (GPS + Galileo) and dual-frequency WAAS user avionics sensor could provide CAT I service to users in most of CONUS, Alaska, and Canada. Importantly, the VPL values are less than 10 m VAL (a more stringent landing requirement) in most of CONUS and Canada, which is a significant improvement. Finally, this paper also presented the analysis results for the similar aviation navigation performance enhancement to Japanese MSAS and European’s EGNOS. The results are equally encouraging.

This paper treated Galileo satellites the same as GPS satellites, but in different orbits, the MAAST simulation results for the dual-mode (GPS + Galileo) and dual-frequency WAAS user avionics sensor might be optimistic. It does not consider and model the time reference difference between GPS and Galileo signals (group delays) and the coordinate difference between these two systems. These differences might have some impact at performance.

MAAST was intended as an efficient and effective tool for algorithm development. It is strictly deterministic, and does not model asset failures in a probabilistic manner. Despite these limitations, the results of this paper show that the performance of WAAS can be dramatically improved with the upgraded system which features new additional civil signal (L5), and a new additional satellites constellation (Galileo).
